# A Case of Radical Resection of Esophageal Basaloid Cell Carcinoma After Hemostasis by Transarterial Embolization

**DOI:** 10.1155/cris/7736573

**Published:** 2025-01-10

**Authors:** Keita Sato, Koji Takahashi

**Affiliations:** Department of Surgery, Ise Red Cross Hospital, 1-471-2 Funae, Ise City, Mie, Japan

**Keywords:** basaloid cell carcinoma, transarterial embolization, upper gastrointestinal bleeding

## Abstract

Esophageal bleeding management typically involves endoscopy but becomes challenging with large or hemorrhagic tumors, especially in cases of rare basal cell carcinoma. This malignancy, with a poorer prognosis than squamous cell carcinoma, often requires definitive surgery. A 78-year-old man with severe hematemesis underwent transarterial embolization (TAE) after failed endoscopic hemostasis for a middle thoracic esophageal tumor. Subsequently, he successfully underwent radical tumor resection on the seventh day of hospitalization. While emergency surgery is an option, its invasiveness may be a limitation, especially for patients in poor general condition. TAE is effective for hemostasis and serves as a crucial bridge to radical esophageal tumor resection.

## 1. Introduction

Traditionally, the first procedure for intraesophageal bleeding is an endoscopic hemostasis, and transarterial embolization (TAE) can be an effective alternative procedure [1]. There are only a few case reports of the use of liquid embolization agents such as gelatin sponges, microcoils, and N-butyl cyanoacrylate (NBCA) as a treatment for esophageal bleeding [2]. Most reported cases of nonvariceal bleeding from the esophagus are due to locally advanced esophageal cancer that has invaded surrounding organs, postoperative bleeding, or ulcerative lesions; bleeding from resectable esophageal cancer is rare. We report a case of esophageal bleeding from preoperative esophageal cancer treated with TAE for hemostasis followed by complete radical resection.

## 2. Case Presentation

**Figure 1:**
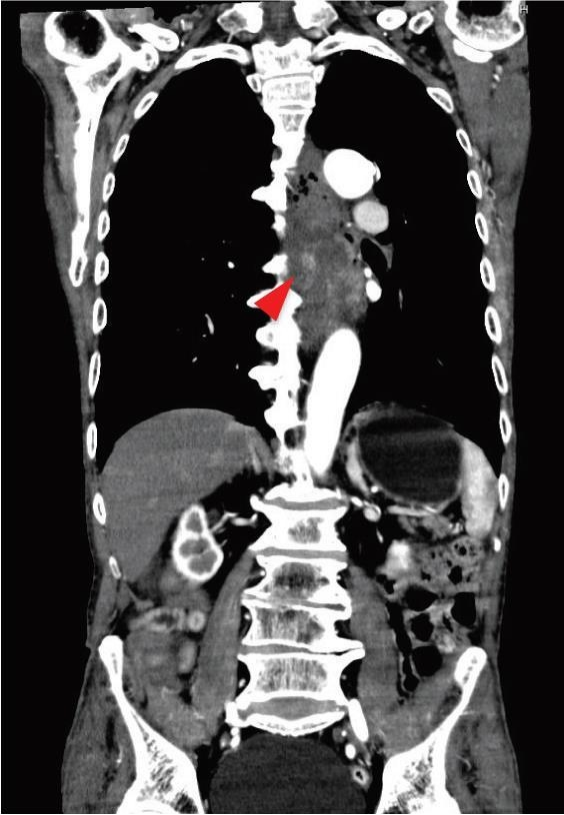
Enhanced computed tomography on arrival. Contrast-enhanced CT showed a large tumor in the middle to lower esophagus. Extravasation of contrast medium toward the esophageal lumen was observed (arrowhead).

**Figure 2:**
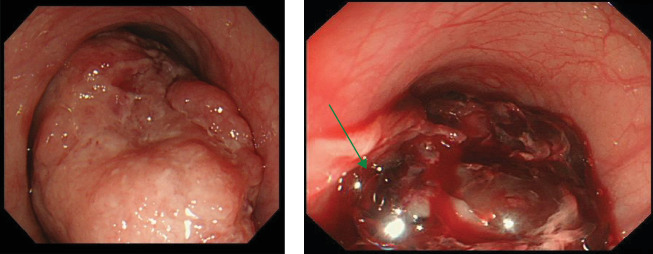
Upper gastroendoscopic images. (a) Endoscopic view of the tumor showed a protruded lesion at the middle thoracic esophagus. (b) Endoscopy showed active bleeding (arrows), but it was difficult to see the bleeding point directly because of the large amount of blood and large size of the tumor.

**Figure 3:**
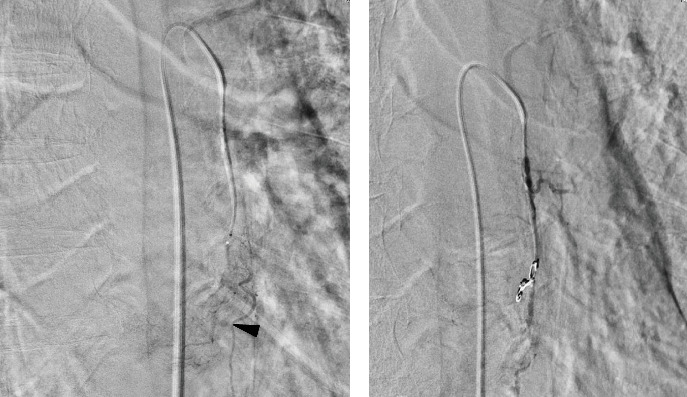
Angiographic images. (a) Selective angiography of proper esophageal artery showed tumor and pseudoaneurysm (arrowhead) in the lower esophagus. (b) The patient was embolized with gelatin sponges and microcoils, and hemostasis was successfully achieved.

A 78-year-old man was in a state of shock due to sudden massive hematemesis after a meal. He had no remarkable medical history and no medication. Contrast-enhanced CT showed an 11 cm large mass lesion in the middle to lower esophagus with extravasation of contrast media (Figure 1). Emergency upper gastrointestinal endoscopy showed an elevated lesion (Figure 2a) and large clot. Even though the bleeding was active, it was difficult to observe due to the large size of the mass and the bleeding point could not be identified (Figure 2b). It was determined that hemostasis by endoscopy would be difficult, and hemostasis by TAE was performed. Selective angiography of the proper esophageal artery revealed a hypervascular tumor and pseudoaneurysm of contrast media in the lower esophagus (arrowhead in Figure 3a). Successful hemostasis was obtained by embolizing the proper esophageal artery directly derived from the aorta with gelatin sponge and microcoils (Figure 3b).

After hemostasis was achieved by TAE, he underwent a subtotal esophagectomy with three-field lymph nodes dissection via right thoracotomy and reconstruction using a gastric tube via the posterior sternal route on day 7 of hospitalization. Around the tumor, especially at the border with the aorta, there was strong adhesion, possibly due to inflammation associated with embolization.

**Figure 4:**
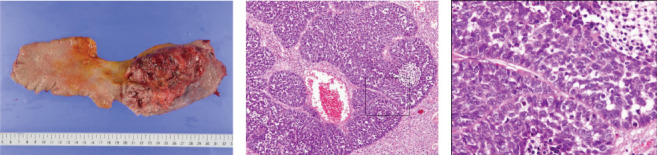
Resected specimen and histopathological examination findings. (a) The lesion was well-demarcated and elevated tumor at lower esophagus and measured 110 × 78 × 52 mm. (b, c) Histopathological examination revealed that basement membrane-like cells were arranged in a fenestrated pattern. (b) HE × 50. (c) HE × 400.

The resected specimen was well-demarcated and elevated tumor and measured 110 × 78 × 52 mm (Figure 4a). Histopathological examination revealed that basement membrane-like cells were arranged in a fenestrated pattern (Figure 4b,c) and led to the diagnosis of basaloid cell carcinoma. The tumor had invaded beyond the muscular mucosae, but there was no exposure to the avulsion surface. The final diagnosis was pT3, pN0, ly0, v1a, pPM0, pDM0, pRM0, M0, and pStage II. His postoperative course was uneventful and was transferred to the hospital on postoperative day 27.

## 3. Discussion

There are few previous reports of TAE for esophageal cancer that is difficult to hemostasis endoscopically. The most common cases of TAE for esophageal lesions are esophageal ulcers, postoperative bleeding, Mallory–Weiss syndrome, and tumor invasion of unresectable esophageal cancer [2–6]. TAE for esophageal cancer, especially preoperative and *resectable* esophageal cancer, is extremely rare [3, 4].

The use of IVR for managing esophageal bleeding is rare, with a total of 22 reported cases including our own upon reviewing past cases (excluding those lacking detailed descriptions) [2–4, 7]. Among these cases (Table 1), bleeding associated with esophageal cancer was the most frequent at 12 cases (including four postoperative bleedings). Of these 12 cases, four were postoperative bleeds, three cases were stage IV, and three cases had unclear staging; only two cases involved resectable lesions, including our own case. These two cases represent exceptionally rare instances where curative resection became possible after hemostasis using IVR.

The clinical success rate of IVR for esophageal bleeding is 90.5% (19/22 cases), with complications including one case each of gastric necrosis and perforation as a result of embolization. Among the documented cases alone, 11 cases exhibited coagulopathy, with embolic materials used including NBCA in 16 cases, gelatin sponge in four cases, and coils in one case. NBCA, a permanent embolic agent, has been reported as safe for upper gastrointestinal bleeding [8], making it a crucial treatment option depending on patient vital signs and coagulation status.

In this case, the significance lies in utilizing IVR to achieve hemostasis and facilitate a curative resection, thereby bridging critical stages of treatment. Particularly, surgery for esophageal cancer involving three-field lymph node dissection is a prolonged and highly invasive procedure compared to other surgeries, often requiring intraoperative repositioning. In cases requiring emergency surgery due to tumor bleeding, as reported, there may be accompanying vital instability and coagulation abnormalities. This necessitates abbreviating surgery to reduce invasiveness by omitting meticulous lymphadenectomy and completing the procedure in a shorter timeframe. This is a serious problem from an oncological point of view.

Consequently, prioritizing hemostasis may potentially prevent achieving curative resection. While cases undergoing deferred surgery after IVR have reported a favorable prognosis of 72%–89% [9], our case further achieved long-term oncological prospects due to successful curative resection.

## 4. Conclusion

Based on our clinical experience, we propose that TAE is a practical and successful method for controlling bleeding in cases of esophageal cancer. Considering the high invasiveness of esophageal cancer surgery and the increased likelihood of complications, especially in patients with compromised overall health, our results highlight the promising role of TAE as an emergency oncologic intervention. TAE not only effectively stops bleeding but also offers a strategic and viable option, paving the way for subsequent radical resection procedures.

## Figures and Tables

**Table 1:** Cases in which IVR was performed for esophageal hemorrhage.

Total	*N* = 22
Diseases	
Esophageal cancer	12
Ulcer	4
Mallory–Weiss	4
Inflammation	2
Embolized vessels	
Left gastric artery	9
Proper esophageal artery	8
Bronchial artery	4
Patient condition	
Shock	11
Coagulopathy	8
Complication	
Re-IVR	2
Gastric infarction perforation	1
Technical success	19 (90.3%)
**IVR for esophageal cancer**	***N*** ** = 12**
State of cancer	
Resectable cancer	2
Unresectable, stage Ⅳ	3
Postoperative bleeding	4
Unknown	3
Prognosis	
Discharged	3
Radical resection	2
RT	1
MOF	2
Died of disease	3
Unknown	1

## Data Availability

We do not have additional data or supporting information files.
